# Cardiac Function Assessment in Fetuses With Ductus Arteriosus Constriction: A Two-Dimensional Echocardiography and FetalHQ Study

**DOI:** 10.3389/fcvm.2022.868675

**Published:** 2022-07-26

**Authors:** Jing Ma, Haiyan Cao, Liu Hong, Juanjuan Liu, Xiaoyan Song, Jiawei Shi, Yi Zhang, Li Cui, Li Zhang, Mingxing Xie

**Affiliations:** ^1^Department of Ultrasound, Union Hospital, Tongji Medical College, Huazhong University of Science and Technology, Wuhan, China; ^2^Clinical Research Center for Medical Imaging in Hubei Province, Wuhan, China; ^3^Hubei Province Key Laboratory of Molecular Imaging, Wuhan, China

**Keywords:** fetal arterial duct constriction, cardiac function, FetalHQ, 24-segment fractional shortening, 24-segment sphericity index

## Abstract

**Background:**

Fetal ductal constriction (DC) is associated with excessive polyphenol-rich food (PRF) consumption during pregnancy. However, the effect of this hemodynamic change on fetal cardiac function still needs to be elucidated. Therefore, this study aimed to evaluate the cardiac function of fetuses with PRF-related DC and to describe serial observations of cardiac function changes.

**Methods:**

We compared the traditional echocardiographic indices, including morphological, hemodynamic, and functional parameters, between study fetuses and controls. For global and segmental deformation analysis of the left and right ventricles, fetalHQ with the speckle-tracking technique was used to calculate sphericity index (SI), global longitudinal strain (GLS), fractional shortening (FS), fractional area change (FAC), etc. In addition, follow-up data were compared with the generalized linear model.

**Results:**

A total of 60 DC fetuses and 60 gestational-matched controls were enrolled in our study, with 20 DC fetuses undertaking a follow-up echocardiogram after 2–3 weeks. Compared with controls, there was a distinct decrease in right ventricular GLS (RVGLS) (−13.39 ± 3.77 vs. −21.59 ± 2.51, *p* < 0.001), RVFAC (22.20 ± 9.56 vs. 36.01 ± 4.84, *p* < 0.001), left ventricular GLS (LVGLS) (−19.52 ± 3.24 vs. −23.81 ± 2.01 *p* < 0.001), and LVFAC (39.64 ± 7.32 vs. 44.89 ± 4.91, *p* = 0.004). For 24-segment FS analysis, DC fetuses showed lower FS in left ventricular (LV) segments 18–24, with no difference in LV segments 1–17. Right ventricular (RV) FS in segments 4–23 was also reduced in the DC group. The 24-segment SI analysis indicated significantly lower SI in DC than those in controls for LV segments 1–14 and RV segments 19–24. We found that the pulsatility index (PI) of ductus arteriosus (DA) was an independent variable for RVGLS (β = −0.29, *p* = 0.04). In 20 DC fetuses with follow-up echocardiograms, no obvious difference in myocardial deformation was found between the initial examination and follow-up data.

**Conclusion:**

Left and right ventricular performances were both impaired in DC fetuses, along with a series of morphological and hemodynamic changes. Although the state of DA constriction improved on second examinations, cardiac function was not completely restored.

## Introduction

Ductus arteriosus (DA) patency is essential for normal hemodynamics *in utero*. Previous studies have indicated that blood flow across the DA accounts for approximately 78% of the right cardiac output and 46% of the combined cardiac output (1). Premature constriction or closure of the DA can occur during fetal life, especially in the third trimester of pregnancy. It may lead to right heart failure, hydrops fetalis, and even intrauterine death in the most severe cases (2). Ductal constriction (DC) is associated with maternal exposure to prostaglandin synthase inhibitors such as non-steroidal anti-inflammatory drugs (NSAIDs) in many published cases. In recent years, excessive polyphenol-rich food (PRF) consumption during pregnancy has also been reported to be associated with DC (3–6). DC causes fetal blood flow redistribution, leading to increased pulmonary artery pressure and pulmonary resistance (7, 8). However, up to now, most studies on PRF-related DC focused mainly on etiology rather than cardiac function change. Hence, the effect of DC on fetal cardiac performance still needs to be elucidated.

Although traditional echocardiographic parameters, such as ejection fraction (EF), fractional area change (FAC), and myocardial performance index (Tei index), are used to evaluate fetal cardiac function in drug-induced DC, it is impossible to specify changes in cardiac function because measurement of these parameters is affected by beam angle, fetal position, and other objective conditions (9). Based on the measurements of global longitudinal strain (GLS) and FAC, FetalHQ divides the ventricle into 24 segments to measure fractional shortening (FS) and sphericity index (SI), and evaluates the transverse myocardial contractility of successive segments (10, 11). The studies on fetal growth restriction and twin-to-twin transfusion syndrome (TTTS) found that the novel technique detailed cardiac shape and function analysis.

Therefore, this study aimed to evaluate the cardiac function of fetuses with polyphenol-induced DC and to make serial observations of the ventricular performance.

## Materials and Methods

### Study Population

**FIGURE 1:**
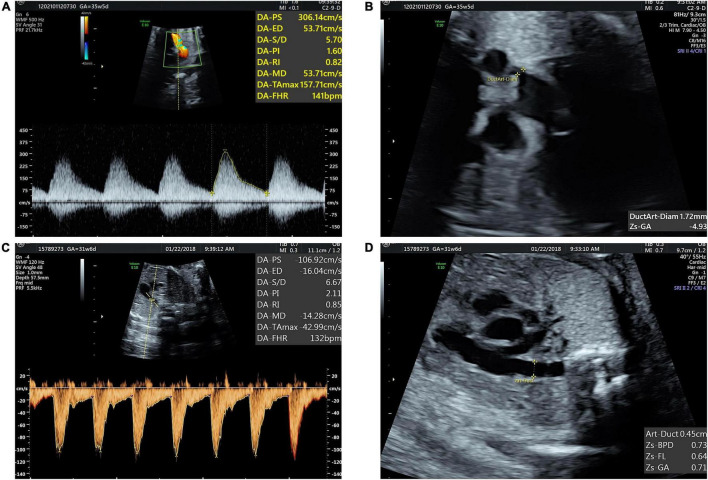
Fetal ductus arteriosus constriction **(A)** blood flow spectrum of ductus arteriosus (DA) showed decreased PI and increased peak systolic and diastolic velocities. **(B)** Three vessels and trachea (3VT) view showed DA narrowed. **(C,D)** Normal blood flow spectrum of DA and tubular-shaped DA.

We retrospectively reviewed 60 fetuses with 24–34 gestational weeks diagnosed as DC by echocardiography from January 2016 to April 2022 in the Department of Ultrasound, Union Hospital, Tongji Medical College, Huazhong University of Science and Technology, Wuhan, China. After the diagnosis of DA constriction, we advised pregnant women to suspend polyphenols and expected them to come back for a follow-up in 2–3 weeks. Follow-up records were collected for 20 fetuses. Sixty normal fetuses with matching gestational age were selected as the control group. The prenatal diagnosis of DC was based on the following ultrasonic signs: (1) DA peak systolic velocity (PSV) ≥ 140 cm/s; (2) DA maximum diastolic velocity ≥ 30 cm/s; and (3) DA pulsatility index (PI) ≤ 2.2. All the three criteria are necessary for DC diagnosis (12) (Figure 1). The exclusion criteria for both the groups included: (1) twin or multiple pregnancies; (2) complicated with other structural heart defects; (3) complicated with severe extracardiac structure malformation; (4) fetal growth restriction; (5) current infection in pregnant women; and (6) other maternal factors affecting fetal hemodynamics, such as gestational hypertension, diabetes, and hyperthyroidism.

### Fetal Echocardiography

A detailed fetal echocardiogram was performed by two experienced fetal echocardiography physicians using Voluson E10 and E8 ultrasound machines with eM6C and C2-9 transducers (2–9 MHz) (GE Healthcare, United States). Conventional views and two-dimensional four-chamber dynamic images were obtained according to a sonographic screening examination of the fetal heart (13). Biventricle end-diastolic transverse diameters, length, foramen ovale (FO) diameter, ascending aorta (AAo) *z*-score, aortic valve (AV) z-score, main pulmonary artery (MPA) *z*-score, pulmonary valve (PV) *z*-score, left pulmonary artery (LPA) *z*-score, right pulmonary artery (RPA) *z*-score, and DA *z*-score were measured. The global sphericity index (GSI) was calculated by 4-chamber view length/4-chamber view width. The fetal heart blood flow, heart rate, and rhythm were observed and evaluated by M-mode echocardiography and color Doppler flow imaging. The spectrum of the aorta, the pulmonary artery, and the ductus arteriosus was obtained by spectral Doppler. The peak systolic velocity of arteriovenous (AV) and PV flow and the peak systolic and diastolic velocity of DA were measured at three vessels and the trachea (3VT) view. The spectrum of DA for PI was manually traced. The acceleration time/ejection time (AT/ET) was calculated from fetal pulmonary artery flow (FPAF) velocity waveforms. The above data were measured repeatedly three times and the results were averaged. The results of fetal echocardiography were blinded to the experimenter.

### Evaluation of Fetal Cardiac Function by FetalHQ

A cardiac cycle was selected from the two-dimensional (2D) 4-chamber images for extracting the parameters of cardiac shape and function. As described in the previous investigation (11), fetalHQ software (GE Healthcare Austria GmbH & Co OG, Voluson™ E10) automatically tracked endocardial border contours from the lateral wall to the apex and from the apex to the base of the septal wall at the end-systolic and end-diastolic time The GLS, the FAC, the 24-segment FS, and 24-segment SI were calculated for both ventricles; EF, cardiac output (CO), and stroke volume (SV) were evaluated for the left ventricle.

### Statistical Analysis

Statistical analysis was performed by SPSS version 23.0 (IBM Incorporation, Armonk, New York, United States) and GraphPad Prism version 9.0.0 (121) for Windows (GraphPad Software, San Diego, California, United States).^1^ Continuous variables were expressed as mean ± SD or median (interquartile range). The Shapiro–Wilk test was used to evaluate the data normality. For normally distributed continuous variables, the Student’s *t*-test for independent samples was used for comparisons between the groups. For those non-normally distributed data, the Mann–Whitney *U*-test was applied. Categorical variables were analyzed by the chi-squared test or Fisher’s exact test when appropriate. The univariate regression analysis was used to identify the risk factors affecting cardiac performance in fetal DC. Follow-up data were compared with the generalized linear model. Left ventricular GLS (LVGLS), LVFAC, right ventricular GLS (RVGLS), and RVFAC of 30 randomly selected fetuses for interobserver and intraobserver reproducibility were calculated by intraclass correlation coefficients (ICCs) with a 95% CI. A *p*-value < 0.05 was considered statistically significant.

## Results

### Baseline Characteristics

The baseline characteristics of the study population are shown in Table 1. DC fetuses and controls were similar in terms of gestational age, maternal age, body mass index (BMI), history of medication, history of infection, and estimated fetal weight (EFW). Of the 60 DC fetuses diagnosed at 30.74 ± 3.76 weeks, maternal age was 29.47 ± 3.47 years. EFW in DC fetuses was 1587.14 ± 665.43 g. The gestational weeks for the initial examination were 28.30 ± 3.27 weeks in normal fetuses and 30.74 ± 3.76 weeks in DC fetuses.

**TABLE 1:** Baseline characteristics of the study population.

Characteristics	Control group (*n* = 60)	DC group (*n* = 60)	*P*-value
Maternal age (years)	28.68 ± 3.46	29.47 ± 3.41	0.33
BMI (kg/m^2^)	25.81 ± 3.42	24.97 ± 2.81	0.70
GA at diagnosis (weeks)	28.30 ± 3.27	30.74 ± 3.76	0.52
**Obstetric history**			
Gravidity	2 (1–3)	2 (1–3)	0.32
Parity	0 (0–1)	1 (0–1)	0.35
Abortion	1 (0–1)	0 (0–1)	0.50
History of medication	7 (11.67%)	11 (18.33%)	0.71
History of infection	18 (30.00%)	12 (20.00%)	0.56
Estimated fetal weight (EFW) (g)	1403.93 ± 540.71	1587.14 ± 665.43	0.21

*Values are expressed as mean ± SD or n (%). BMI, body mass index; GA, gestational weeks.*

### Conventional Echocardiography Results

Fetal cardiac morphometric and hemodynamic measurements are given in Table 2. As expected, DC presented constricted dimension, accelerated velocity, and decreased PI in DC fetuses. The mean RV/LV width ratio for DC fetuses [1.18 (1.00–1.41) vs. 1.06 (1.01–1.11), *p* = 0.006] was enlarged. No obvious disproportion in great arteries (similar MPA/AO diameter ratio) was detected. The aortic and pulmonary dimensions did not differ between the groups, except for the slightly lower diameter of the pulmonary valve annulus in DC [−0.18 (−0.53 to 0.26) vs. 0.28 (−0.05 to 0.54), *p* = 0.01]. DA z-score [−1.23 (−2.24 to −0.65) vs. 0.16 (−0.14 to 0.75), *p* < 0.001] was lower in the DC group. Blood flow across the aortic and pulmonary valves was not accelerated or decelerated when compared with controls. Fetuses with DC showed a decrease in pulmonary AT/ET ratio (0.20 ± 0.04 vs. 0.23 ± 0.04, *p* = 0.02). Tricuspid regurgitation occurred in 70% of fetuses, including mild, moderate, and severe regurgitation in 28 (46.67%), 8 (13.33%), and 6 (10.00%) fetuses.

**TABLE 2:** Fetal conventional echocardiographic parameters in the study population.

Variable	Control group (*n* = 60)	DC group (*n* = 60)	*P*-value
**Morphometric measurements**			
GSI	1.41 ± 0.15	1.27 ± 0.13	0.002
RV/LV width ratio	1.06 (1.01–1.11)	1.18 (1.00–1.41)	0.006
Cardio-thoracic ratio	0.56 (0.53–0.58)	0.58 (0.54–0.63)	0.028
DA *z*-score	0.16 (−0.14 to 0.75)	−1.23 (−2.24 to −0.65)	<0.001
Pulmonary valve *z*-score	−0.18 (−0.53 to 0.26)	0.28 (−0.05 to 0.54)	0.01
Main PA *z*-score	0.73 (0.25–1.07)	0.60 (0.24–1.00)	0.86
LPA *z*-score	0.13 (−0.16 to −0.51)	0.26 (−0.21 to 0.46)	0.99
RPA *z*-score	0.05 (−0.30 to 0.39)	0.09 (−0.45 to 0.45)	0.78
AV *z*-score	0.14 (−0.45 to 0.47)	0.06 (−0.61 to 0.52)	0.81
AA *z*-score	0.01 (−0.25 to 0.51)	0.21 (−0.16 to 0.50)	0.61
MPA/AAo	1.27 ± 0.11	1.30 ± 0.17	0.74
FO (cm)	0.53 ± 0.06	0.58 ± 0.16	0.34
**Hemodynamic measurements**			
**Pulmonary artery**			
Peak systolic velocity (cm/s)	77.56 ± 10.21	72.38 ± 16.53	0.13
Acceleration time (AT) (s)	43.33 ± 6.70	40.40 ± 10.53	0.25
Ejection time (ET) (s)	189.53 ± 16.73	203.91 ± 30.06	0.04
AT/ET	0.23 ± 0.04	0.20 ± 0.04	0.02
**Ductus ateriosus**			
Peak systolic velocity (cm/s)	102.65 ± 15.57	172.07 ± 39.77	<0.001
Peak diastolic velocity (cm/s)	18.54 ± 4.53	41.04 ± 13.82	<0.001
Pulsatility index	2.72 ± 0.29	1.89 ± 0.47	<0.001
**Ascending aorta**			
Peak systolic velocity (cm/s)	101.67 ± 9.71	104.44 ± 30.03	0.24
Tricuspid regurgitation			
Mild		28 (46.67%)	
Moderate		8 (13.33%)	
Severe		6 (10.00%)	
FHR (BMP)	144 ± 8.00	146 ± 10.00	0.6

*Values are expressed as mean ± SD or median (interquartile range). AAo, ascending aorta; AV, aortic valve; MPA, main pulmonary artery; PV, pulmonary valve; LPA, left pulmonary artery; RPA, right pulmonary artery; DA, ductus ateriosus; FO, foramen ovale; GSI, global sphericity index.*

### Fetal Cardiac Functional Results

Fetal cardiac functional measurements of the study population are given in Table 3. There was a distinct decrease in RVGLS (−13.39 ± 3.77 vs. −21.59 ± 2.51, *p* < 0.001), RVFAC (22.20 ± 9.56 vs. 36.01 ± 4.84, *p* < 0.001), LVGLS (−19.52 ± 3.24 vs. −23.81 ± 2.01, *p* < 0.001), and LVFAC (39.64 ± 7.32 vs. 44.89 ± 4.91, *p* = 0.004). The stroke volume (SV), cardiac output (CO), and CO by EFW were similar between the two groups.

**TABLE 3:** Fetal functional echocardiographic parameters in the study population.

Variable	Control group (*n* = 60)	DC group (*n* = 60)	*P*-value
**LV function**			
EF (%)	59.13 ± 6.01	54.65 ± 10.99	0.08
FAC (%)	44.89 ± 4.91	39.64 ± 7.32	0.004
SV (ml)	0.89 ± 0.29	1.02 ± 0.63	0.87
CO (ml/min)	125.5 ± 41.91	137.78 ± 76.78	0.74
CO by EFW (ml/min/kg)	95.20 ± 39.97	94.68 ± 43.42	0.64
GLS (%)	−23.81 ± 2.01	−19.52 ± 3.24	<0.001
**RV function**			
FAC (%)	36.01 ± 4.84	22.20 ± 9.56	<0.001
GLS (%)	−21.59 ± 2.51	−13.39 ± 3.77	<0.001

*Values are expressed as mean ± SD. GLS, global longitudinal strain; FAC, fractional area change; EF, ejection fraction; CO, cardiac output; SV, stroke volume.*

**FIGURE 2:**
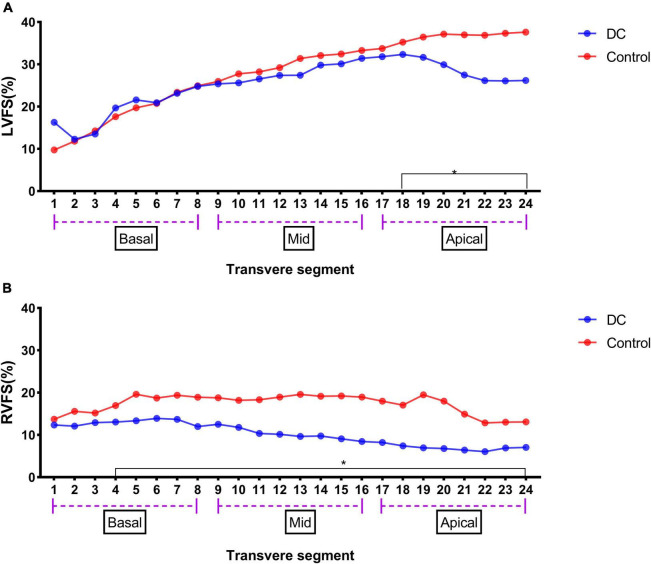
LV 24-segment FS in the DA constriction group and the control group **(A)** and RV 24-segment FS in the DA constriction group and the control group **(B)**. *p < 0.05 compared in DC group and control group.

The results of the 24-segment FS analysis of LV and RV are shown in Table 4. There was no significant difference in the LVFS (%) from segments 1 to 17 between the DA constriction group and the normal group, but the LVFS values from segments 18 to 24 decreased. The RVFS (%) from segments 4 to 23 was lower than that in the normal group (Figure 2).

**TABLE 4:** Biventricles 24-segments FS in the DC group and the control group.

Variable	Control group (*n* = 60)	DC group (*n* = 60)	*P*-value
**LVFS (%)**			
Segment 1	13.16 (3.52–21.73)	16.82 (3.64–28.33)	0.341
Segment 2	15.12 (7.5–22.85)	11.02 (0.52–23.03)	0.315
Segment 3	18.1 (11.1–24.16)	14.27 (2.76–24.16)	0.294
Segment 4	19.22 (13.46–25.82)	22.22 (14.35–30.26)	0.157
Segment 5	20.61 (15.13–28.03)	23.95 (16.07–31.19)	0.222
Segment 6	22.22 (18.23–30.38)	23.34 (15.24–29.9)	0.990
Segment 7	23.88 (18.77–30.16)	24.99 (15.89–31.08)	0.744
Segment 8	26.31 (20.87–31.78)	23.94 (14.4–31.27)	0.125
Segment 9	26.52 (22.31–33.08)	24.09 (14.98–32.43)	0.393
Segment 10	28.38 (24.12–34.47)	24.31 (14.9–33.01)	0.181
Segment 11	30.1 (26.13–35.74)	24.55 (15.65–34.73)	0.107
Segment 12	32.14 (27.54–36.99)	23.54 (17.33–35.41)	0.063
Segment 13	32.88 (28.35–38.86)	23.61 (19.13–35.81)	0.058
Segment 14	33.41 (30.02–40.57)	24.32 (19.25–36.92)	0.061
Segment 15	33.71 (31.14–40.96)	25.62 (21.06–36.91)	0.080
Segment 16	34.91 (31.06–42.54)	26.93 (21.21–37.13)	0.071
Segment 17	35.97 (30.86–43.58)	27.44 (20.82–37.03)	0.393
Segment 18	36.72 (31.52–45.21)	27.03 (19.94–37.78)	<0.001
Segment 19	38.64 (32.09–46.9)	26.91 (16.01–35.68)	<0.001
Segment 20	41.58 (32.79–48.06)	26.36 (14.63–36.49)	<0.001
Segment 21	41.57 (32.8–48.77)	25.34 (12.7–36.04)	<0.001
Segment 22	41.75 (33.09–48.79)	25.03 (10.29–36.01)	<0.001
Segment 23	41.68 (33.29–48.95)	24.03 (9.47–36.63)	<0.001
Segment 24	41.65 (33.42–49.43)	23.73 (8.36–36.68)	<0.001
**RVFS (%)**			
Segment 1	13.77 (2.89–23.26)	10.85 (−1.26 to 22.9)	0.427
Segment 2	15.77 (6.38–23.53)	10.2 (0.74–22.93)	0.200
Segment 3	16.28 (10.07–23.34)	11.23 (1.55–22.63)	0.085
Segment 4	18.23 (10.78–22.91)	12.28 (2.41–21.56)	0.032
Segment 5	19.12 (11.9–23.25)	12.79 (3.78–20.52)	0.010
Segment 6	19.86 (12.72–24.47)	10.87 (3.46–16.83)	<0.001
Segment 7	20.42 (13.58–24.23)	10.43 (2.45–16.14)	<0.001
Segment 8	20.12 (13.93–24.37)	9.48 (2.77–17.28)	<0.001
Segment 9	20.07 (15.03–25.04)	9.17 (2.27–17.4)	<0.001
Segment 10	20.53 (15.17–25.67)	8.86 (2.06–17.53)	<0.001
Segment 11	20.99 (15.35–26.95)	8.57 (2.24–16.64)	<0.001
Segment 12	20.56 (15.82–27.52)	8.77 (2.02–16.46)	<0.001
Segment 13	21.34 (15.06–28.41)	8.23 (1.59–16.21)	<0.001
Segment 14	22.56 (13.51–29.14)	7.74 (0.66–16.31)	<0.001
Segment 15	22.23 (11.79–29.89)	7.58 (0.43–15.61)	<0.001
Segment 16	22.12 (11.6–29.5)	7.37 (−0.77 to 15.42)	<0.001
Segment 17	21.64 (11.48–28.93)	6.97 (−3.38 to 15.05)	<0.001
Segment 18	21.8 (10.15–26.41)	5.39 (–3.07–14.21)	<0.001
Segment 19	19.83 (7.54–26.13)	4.78 (−1.92 to 14.43)	<0.001
Segment 20	18.23 (5.34–25.09)	5.33 (−3.52 to 14.67)	<0.001
Segment 21	15.71 (2.7–25.23)	4.02 (−4.52 to 14.32)	0.002
Segment 22	14.9 (1.32–24.69)	3.79 (−3.51 to 14.81)	0.004
Segment 23	14.3 (1.27–24.76)	4.98 (−2.61 to 14.64)	0.009
Segment 24	13.88 (1.74–24.8)	4.41 (−4.34 to 13.13)	0.013

**FIGURE 3:**
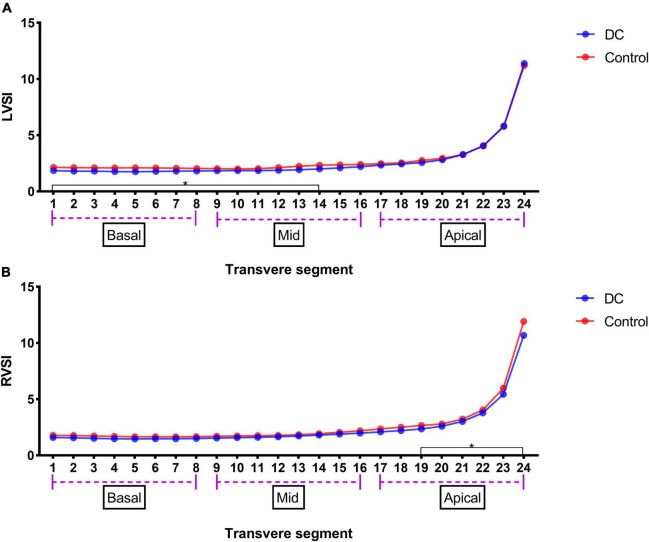
LV 24-segments SI in the premature DA group and the control group **(A)** and RV 24-segments SI in the premature DA group and the control group **(B)**. *p < 0.05 compared in DC group and control group.

The results of the 24-segment SI analysis of LV and RV are shown in Table 5. LVSI from segments 1–14 was lower in the DC group than that in the control group, but RVSI began to significantly decrease from segment 19 (Figure 3).

**TABLE 5:** Biventricles 24-segments SI in the DA constriction group and the control group.

Variable	Control group (*n* = 60)	DC group (*n* = 60)	*P*-value
**LVSI**			
Segment 1	2.12 (1.65–2.42)	1.79 (1.32–2.24)	0.040
Segment 2	2.04 (1.66–2.4)	1.75 (1.36–2.16)	0.033
Segment 3	1.97 (1.65–2.31)	1.71 (1.38–2.09)	0.032
Segment 4	1.95 (1.65–2.28)	1.77 (1.63–2.04)	0.040
Segment 5	1.94 (1.63–2.29)	1.77 (1.62–1.99)	0.038
Segment 6	1.93 (1.62–2.29)	1.81 (1.61–1.96)	0.039
Segment 7	1.94 (1.63–2.31)	1.84 (1.63–1.97)	0.031
Segment 8	1.95 (1.65–2.33)	1.83 (1.61–2.04)	0.032
Segment 9	2.01 (1.68–2.35)	1.84 (1.62–2.09)	0.033
Segment 10	2.01 (1.7–2.35)	1.85 (1.65–2.12)	0.033
Segment 11	2.02 (1.73–2.38)	1.86 (1.67–2.15)	0.029
Segment 12	2.1 (1.75–2.41)	1.9 (1.67–2.18)	0.034
Segment 13	2.14 (1.77–2.45)	1.97 (1.75–2.26)	0.041
Segment 14	2.21 (1.81–2.52)	2.06 (1.82–2.32)	0.045
Segment 15	2.28 (1.84–2.6)	2.1 (1.85–2.43)	0.072
Segment 16	2.37 (1.9–2.71)	2.2 (1.94–2.5)	0.098
Segment 17	2.38 (2.01–2.82)	2.32 (2.06–2.56)	0.148
Segment 18	2.52 (2.08–2.89)	2.44 (2.17–2.66)	0.137
Segment 19	2.61 (2.2–3.04)	2.54 (2.26–2.73)	0.177
Segment 20	2.76 (2.36–3.23)	2.78 (2.32–2.96)	0.347
Segment 21	3.16 (2.69–3.69)	3.19 (2.45–3.45)	0.490
Segment 22	3.89 (3.28–4.72)	4.01 (2.89–4.41)	0.568
Segment 23	5.51 (4.65–6.84)	5.77 (4.15–6.39)	0.656
Segment 24	10.67 (8.98–13.25)	11.22 (8.07–12.55)	0.691
**RVSI**			
Segment 1	1.71 (1.46–1.99)	1.69 (1.49–2.03)	0.851
Segment 2	1.68 (1.43–1.92)	1.63 (1.47–1.92)	0.805
Segment 3	1.63 (1.41–1.88)	1.59 (1.43–1.83)	0.883
Segment 4	1.61 (1.37–1.88)	1.6 (1.42–1.77)	0.856
Segment 5	1.6 (1.37–1.88)	1.57 (1.4–1.74)	0.903
Segment 6	1.61 (1.37–1.87)	1.51 (1.37–1.69)	0.185
Segment 7	1.61 (1.38–1.88)	1.52 (1.38–1.69)	0.157
Segment 8	1.62 (1.41–1.91)	1.56 (1.33–1.7)	0.074
Segment 9	1.62 (1.45–1.96)	1.61 (1.34–1.75)	0.08
Segment 10	1.65 (1.5–1.98)	1.65 (1.35–1.8)	0.081
Segment 11	1.7 (1.57–2.03)	1.65 (1.38–1.87)	0.065
Segment 12	1.77 (1.6–2.08)	1.67 (1.43–1.94)	0.059
Segment 13	1.84 (1.64–2.15)	1.73 (1.47–2)	0.063
Segment 14	1.95 (1.71–2.26)	1.8 (1.5–2.09)	0.067
Segment 15	2.06 (1.8–2.36)	1.89 (1.56–2.2)	0.072
Segment 16	2.18 (1.88–2.48)	1.98 (1.66–2.31)	0.063
Segment 17	2.33 (1.98–2.63)	2.09 (1.77–2.36)	0.025
Segment 18	2.44 (2.12–2.84)	2.2 (1.89–2.49)	0.016
Segment 19	2.64 (2.26–3.16)	2.33 (1.98–2.66)	0.009
Segment 20	2.9 (2.43–3.61)	2.56 (2.2–2.93)	0.006
Segment 21	3.33 (2.88–4.33)	2.89 (2.56–3.44)	0.006
Segment 22	4.11 (3.56–5.35)	3.61 (3.19–4.29)	0.005
Segment 23	5.99 (5.05–7.81)	5.16 (4.55–6.14)	0.003
Segment 24	1.71 (1.46–1.99)	1.69 (1.49–2.03)	0.851

### Risk Factors for Fetal Cardiac Deformation

**FIGURE 4:**
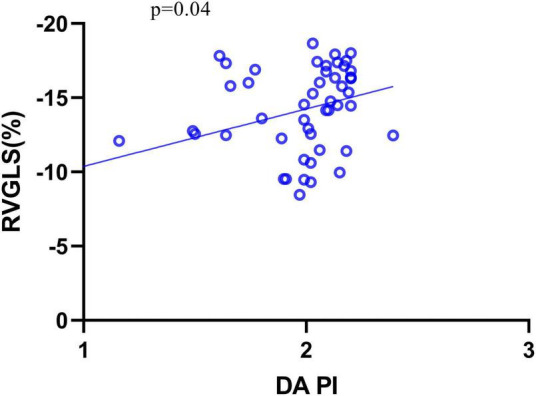
The relationship between RVGLS and DA PI. GLS, global longitudinal strain; PI, pulsatility index.

The correlations between these variables and fetal cardiac deformation (GLS for LV and RV) in DC fetuses were examined using the univariate regression analysis (Table 6), which revealed that only PI of DA was significantly correlated with RVGLS (β = −0.29, *P* = 0.04) (Figure 4).

**TABLE 6:** The univariate regression analysis for RVGLS and LVGLS in the premature DC group.

	LV GLS (%)		RVGLS (%)	
Variable	β	*p*-value	β	*p*-value
GA (weeks)	−0.15	0.28	0.13	0.42
DA/PV	0.19	0.26	−0.33	0.06
PV *z*-score	−0.19	0.29	−0.07	0.68
DA *z*-score	0.30	0.06	−0.29	0.06
ET (s)	−0.21	0.19	0.01	0.96
AT/ET	0.13	0.41	0.05	0.77
Tricuspid regurgitation	−0.17	0.91	0.13	0.26
GSI	0.13	0.44	0.07	0.68
Cardio-thoracic ratio	0.01	0.93	0.31	0.06
DA PS (cm/s)	0.15	0.30	0.10	0.48
DA peak diastolic velocity (cm/s)	0.13	0.42	0.13	0.40
DA PI	−0.06	0.71	−0.29*	0.04

Values are expressed as mean ± SD or median (interquartile range). AAo, ascending aorta; AV, aortic valve; MPA, main pulmonary artery; PV, pulmonary valve; LPA, left pulmonary artery; RPA, right pulmonary artery; DA, ductus ateriosus; GSI, global sphericity index; PS, peak systolic velocity; PI, pulsatility index; AT, acceleration time; ET, ejection time. *p < 0.05.

### Follow-Up Observations

**FIGURE 5:**
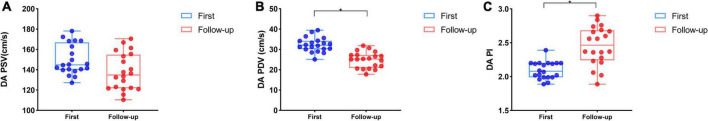
Series examinations of DA flow spectrum for DC fetuses (Blue box and plots represent the first examination in the DC group, red box and plots represent follow-up data in the DC group). At follow-up, DA PSV had no significant difference compared with the initial examination **(A)**, but DA PDV **(B)** and DA PI **(C)** were significantly decreased in the DC group. *p < 0.05 compared first and follow-up examinations. PS, peak systolic velocity (cm/s); PI, pulsatility index; PDV, peak diastolic velocity (cm/s). DC, ductal constriction.

**FIGURE 6:**
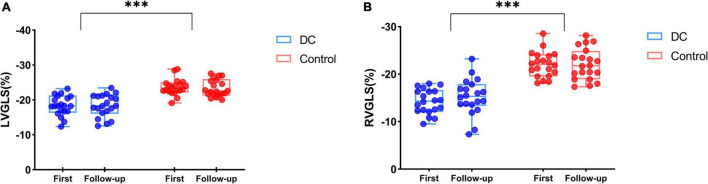
The biventricular function of DC fetuses for follow-up examinations (Blue box and plots represent the GLS in the DC group, red box and plots represent GLS in the control group). Both LVGLS **(A)** and RVGLS **(B)** decreased in the DC group compared with the control group, but no difference was observed at follow-up in DC fetuses. ***p < 0.001, compared in DC group and control group. GLS, global longitudinal strain; DC, ductal constriction.

Twenty (20/60, 33.33%) fetuses in the DC group underwent an echocardiogram two times, first at diagnosis and second after 2–3 weeks of observation. Follow-up data were reviewed with the generalized linear model tests. As expected, during the DC reversal in the follow-up examination, DA peak diastolic velocity (PDV) was significantly decreased and DA PI increased (Table 7 and Figure 5). Myocardial deformation in DC fetuses was lower than that in normal fetuses in the follow-up examination. For the DC group, no significant differences in LVGLS, RVGLS, LVFAC or RVFAC were found between the first and follow-up examination [(Table 8) and (Figure 6)]. Also, gestational age had no effect on myocardial deformation in both groups.

**TABLE 7:** Series examinations of DA constriction fetuses with two-dimensional echocardiography and Doppler measurements.

	Control group (*n* = 20)		DC group (*n* = 20)		Group	Follow-up	Group interact follow-up
Variable	First	Follow-up	First	Follow-up			
** Morphometric measurements**							
FO (cm)	0.46 (0.4–0.52)	0.58 (0.51-0.64)	0.51 (0.45–0.59)	0.64 (0.58–0.68)	<0.001	0.61	0.66
PV *z*-score	0.52 (0.38–0.95)	0.26 (−0.01 to 0.8)	−0.14 (−0.4 to 0.59)	0.07 (−0.3 to 0.35)	<0.001	0.27	0.64
MPA *z*-score	0.8 (0.5–0.96)	0.63 (0.38–1.01)	0.45 (−0.05 to 0.74)	0.64 (0.28–1.09)	0.94	0.53	0.48
LPA *z*-score	0.45 (0.04 to 0.7)	0.37 (0.15–0.89)	−0.07 (−0.51 to 0.47)	0.38 (−0.22 to 0.48)	<0.01	0.52	0.57
RPA *z*-score	0.28 (−0.16 to 0.82)	0.42 (0.06–0.59)	−0.09 (−0.29 to 0.22)	0 (−0.32 to 0.37)	0.13	0.76	0.67
DA *z*-score	0.13 (−0.21 to 0.45)	0.15 (−0.24 to 0.36)	−1.75 (−2.04 to −0.13)	−0.1 (−0.63 to 0.02)	<0.01	0.23	0.13
AAo *z*-score	0.45 (0.11–0.71)	0.28 (−0.2 to 0.5)	0.01 (−0.68 to 0.68)	0.16 (−0.36 to 0.63)	0.35	0.09	0.08
AV *z*-score	0.62 (−0.01 to 0.82)	0.46 (−0.07 to 0.74)	−0.31 (−0.58 to 0.36)	0.11 (−0.56 to 0.6)	0.04	0.88	0.96
** Hemodynamic measurements**							
PV_*V Max*_ (cm/s)	73.37 ± 12.64	74.27 ± 8.99	94.16 (75–98.38)	82.45 (79.71–97.14)	0.31	0.78	0.99
AT (s)	43.64 ± 6.70	42.42 ± 7.56	38.9 ± 12.1	44.3 ± 10.75	0.11	0.86	0.89
ET (s)	184.28 ± 12.17	193.75 ± 18.71	203.1 ± 28.31	203 ± 26.94	<0.01	0.03	0.05
AT/ET	0.23 ± 0.04	0.22 ± 0.03	0.20 ± 0.03	0.20 ± 0.04	0.97	0.15	0.21
DA PS (cm/s)	95.21 ± 9.29	103.36 ± 16.04	148.9 ± 15.87	142.2 ± 20	<0.001	0.67	0.83
DA peak diastolic velocity (cm/s)	18.21 ± 4.15	15.83 ± 6.00	31.8 ± 3.26	24.9 ± 4.19	<0.002	0.79	0.03
DA PI	2.70 ± 0.34	2.56 ± 0.47	2.1 ± 0.13	2.5 ± 0.31	<0.003	0.24	0.02

*Values are expressed as mean ± SD or median (interquartile range). AAo, ascending aorta; AV, aortic valve; MPA, main pulmonary artery; PV, pulmonary valve; LPA, left pulmonary artery; RPA, right pulmonary artery; DA, ductus ateriosus; GSI, global sphericity index; PS, peak systolic velocity; PDV, peak diastolic velocity (cm/s); PI, pulsatility index; AT, acceleration time; ET, ejection time.*

**TABLE 8:** Series examinations in DA constriction fetuses for cardiac function analysis by FetalHQ.

	Control group (*n* = 20)		DC group (*n* = 20)		Group	Follow-up	Group interact follow-up
Variable	First	Follow-up	First	Follow-up			
**LV function**							
LV GLS (%)	−23.87 ± 2.78	−23.92 ± 2.61	−18.57 ± 3.05	−19.31 ± 2.99	<0.001	0.61	0.66
LV FAC (%)	46.37 ± 6.98	47.11 ± 4.91	40.62 ± 7.34	41.32 ± 6.81	0.003	0.67	0.97
LV EF (%)	61.86 ± 9.14	63.79 ± 4.99	55.81 ± 10.93	56.15 ± 8.69	0.01	0.56	0.62
LV SV (ml)	0.38 ± 0.15	0.64 ± 0.23	0.52 ± 0.25	0.82 ± 0.34	0.31	0.08	0.88
LV SV/KG (ml/kg)	0.56 ± 0.18	0.63 ± 0.20	0.51 ± 0.25	0.56 ± 0.31	0.18	0.44	0.59
LV CO (ml/min)	56.7 ± 21.32	95.19 ± 40.13	70.58 ± 36.52	109.91 ± 60.57	0.25	0.001	0.02
LV CO/KG (ml/min/kg)	83.82 ± 26.96	93.31 ± 34.15	69.65 ± 38.02	74.04 ± 53.48	0.06	0.57	0.52
**RV function**							
RV GLS (%)	−23.02 ± 2.46	−23.59 ± 3.07	−14.28 ± 2.65	−15.67 ± 4.25	<0.001	0.27	0.64
RV FAC (%)	39.65 ± 4.94	41.98 ± 7.26	25.34 ± 6.17	28.91 ± 7.38	<0.001	0.06	0.72

*Values are expressed as mean ± SD. GLS, global longitudinal strain; FAC, fractional area change; EF, ejection fraction; CO, cardiac output; SV, stroke volume.*

### Intraobserver and Interobserver Variability

**FIGURE 7:**
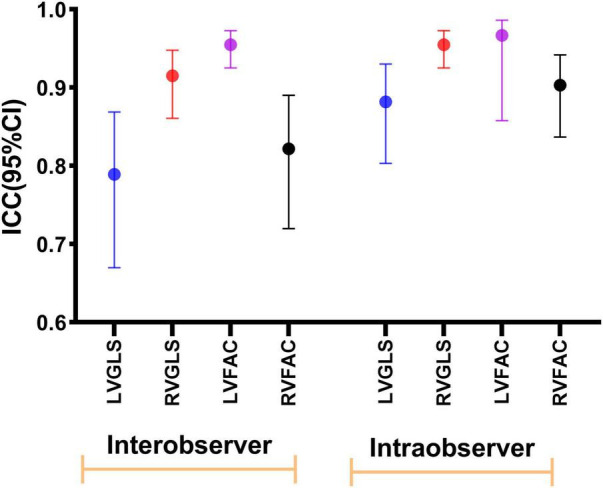
Intraobserver and interobserver reproducibility of LVGLS, RVGLS, LVFAC, and RVFAC represented as ICCs and 95% CIs.

The intraobserver and interobserver intraclass correlation data for LVGLS, LVFAC, RVGLS, and RVFAC indicated excellent results (Figure 7).

## Discussion

Our study found that fetal hearts with DA constriction showed ventricular remodeling and cardiac dysfunction through conventional ultrasound parameters and fetalHQ technology. In normal fetuses, the DA flows in a tubular shape from the distal pulmonary artery branches into the descending aorta, slightly wider than the pulmonary artery; the middle layer of the DA is composed of a large number of smooth muscle cells; it provides an anatomical basis for DA contraction and relaxation (14). DA z-score of DA constriction is significantly lower than that of the normal fetuses, which may be due to maternal factors, such as the consumption of PRF, which could reduce substances maintaining the opening of the DA in fetal circulation, resulting in narrowing of the DA inner diameter and accelerating blood velocity (15–17). Doppler ultrasound was used to measure pulmonary artery blood flow acceleration time (AT) and ejection time (ET), and it was found that the AT/ET ratio reduced in the DA constriction group. Previous studies proved that, in the fetus, the mean pulmonary artery pressure is inversely proportional to the AT/ET ratio, which indirectly illustrated that premature DA constriction would cause higher fetal pulmonary artery pressure, increased pulmonary vascular resistance, and increased afterload of the right heart (18, 19). Pulmonary artery stress increases pressure on the pulmonary valve, resulting in an increased *z*-score of the diameter of the pulmonary valve annulus. If it deteriorates, the result might be pulmonary valve annular enlargement and pulmonary valve regurgitation (2, 3).

Although the difference was statistically significant from segment 19, RV 24-segment SI values were lower than those of normal fetuses. It was also indicated that the right ventricle of fetuses with DA constriction was more globular. Reduced RVGLS, RVFAC, and 24-segment FS values suggest decreased right ventricular compliance and systolic dysfunction. Similar to our results, a preliminary study found that DA constriction led to decreased RVFAC and an increased TEI index (20, 21). The results of the univariate regression analysis indicated that, with the decrease of DA PI value, RVGLS also decreased. As DA PSV and PDV increased with gestational weeks, DA PI could better reflect the situation of DA resistance. Therefore, it further indicated that the aggravation of right heart afterload caused by increased DA resistance was the main cause of the reduction of right heart function (22, 23). Reduced LVGLS and LVFAC suggested left ventricular dysfunction and left ventricular remodeling in DA constriction fetuses. LVFS decreased from segment 18 and SI values also showed a downward trend compared with normal fetuses. However, different from the right ventricle, the decrease of SI values in the left ventricle was mainly found in the basal and middle segments, while the decrease of FS was mainly found in the apex of the left ventricle. Consequently, the ventricular filling function was impaired in the basal and middle parts of the left ventricle, while the systolic function in the apex of the left ventricle was mainly impaired. Because of the special prenatal hemodynamics, left and right ventricular blood flow transportation has two ways, namely, patent foramen right-to-left shunt and DA to the descending aorta through pulmonary artery blood perfusion. Previous animal experiments found that DA constriction caused the left cardiac output to increase, but there was no increase in patent foramen blood flow. Therefore, left heart dysfunction is mainly caused by increased pulmonary blood flow due to decreased right heart output and increased blood flow to the left heart through pulmonary veins, resulting in increased left heart preload. The left ventricle recoils the circumferential fiber in the midwall during the diastolic period, but only the helix fiber is found in the apex of the heart. Only horizontal fibers in the base and the middle can dominate in a clockwise motion when there is an increase in venous return. Therefore, from the perspective of anatomy, there is a possibility of no SI value change in the apex. Increased SI values in the basal and middle segments may be a compensatory mechanism for keeping normal measured FS values and maintaining normal systolic function (24, 25).

We collected the prenatal results of the electrocardiogram of 20 fetuses, during which premature ductal contraction was diagnosed. By comparing the ultrasound parameters of the fetuses in the two examinations and the fetalHQ parameters, we found that the flow rate in the diastolic phase decreased and the PI value increased, but there was no significant change in the systolic flow rate, which might be due to diet control that eased premature contraction (15, 26). RVGLS and RVFAC compared with the initial examination had no obvious differences; this suggests the reversal of DA constriction, but the impact on ventricular function may exist persistently. Our results were different from those of previous studies, in which Harada et al. used Doppler parameters to evaluate the cardiac function of fetuses with premature contraction and found that the diameter of tricuspid annnulus in fetuses increased with premature contraction and decreased after NSAIDs were stopped. Another follow-up study of 45 cases of drug-induced premature catheter contraction found that fetal echocardiography returned to normal within 2–15 days after drug withdrawal (27). However, left ventricular function still had no significant alteration in our study, which may be related to different causes of DA constriction. Previous studies mainly focused on drug-induced premature arterial duct contraction. The subjects in our study, however, had no NASID-taking histories; most of them ate a lot of PRF, which was considered to be the cause of fetal DA constriction. The mechanism of DA constriction induced by polyphenols is different from DA constriction induced by drugs. In addition to reducing maternal prostaglandin E2 (PGE2) by inhibiting COX1 and COX2, polyphenols also participate in antioxidant reactions and indirectly cause maternal PGE2 reduction (17, 28). Another possible reason is that myocardial strain parameters are more sensitive than conventional echocardiography.

In this study, fetal cardiac function was evaluated by the fetalHQ technology for the first time in the setting of DC. Fetal cardiac function contraction mode was described by a 24-segment analysis. Both the left and right ventricles of the fetus with DC were abnormal. In addition to the GLS, the transverse contractility also changed. However, the causes of fetal cardiac function changes are different. The principal cause of right ventricular dysfunction is the increase of ventricular afterload, while left ventricular dysfunction is the result of the secondary increase of ventricular preload. Although premature contraction eased on the second ultrasound examination, cardiac function was not completely restored. Therefore, DA constriction induced by polyphenols requires a close monitoring of changes in fetal cardiac function.

There are some limitations to our study. First, it was a retrospective investigation and single-center design; follow-up data could not be collected for all the fetuses. Second, it was difficult to perform fetal echocardiography in the late trimester of pregnancy; the duration of the effect of premature ductus arteriosus constriction on fetal cardiac function requires further clarification. Finally, we did not measure changes in the flow of the foramen ovale in the state of DA constriction to find out the mechanism responsible for changes in the left cardiac preload.

## Data Availability Statement

The raw data supporting the conclusions of this article will be made available by the authors, without undue reservation.

## Author Contributions

JM performed the experiments and wrote the manuscript. HC performed the data analyses and manuscript preparation. LH and JL helped perform the analysis with constructive discussions. XS and JS contributed to the conception of the study. YZ and LC helped collect follow-up data. LZ and MX provided conception and final approval of the article. All authors contributed to the article and approved the submitted version.

## Conflict of Interest

The authors declare that the research was conducted in the absence of any commercial or financial relationships that could be construed as a potential conflict of interest.

## Publisher’s Note

All claims expressed in this article are solely those of the authors and do not necessarily represent those of their affiliated organizations, or those of the publisher, the editors and the reviewers. Any product that may be evaluated in this article, or claim that may be made by its manufacturer, is not guaranteed or endorsed by the publisher.

## Footnotes

1
www.graphpad.com
